# Evaluation of polyherbal formulation and synthetic choline chloride on choline deficiency model in broilers: implications on zootechnical parameters, serum biochemistry and liver histopathology

**DOI:** 10.5713/ajas.18.0018

**Published:** 2018-04-12

**Authors:** Ramasamy Selvam, Marimuthu Saravanakumar, Subramaniyam Suresh, CV Chandrasekeran, D’Souza Prashanth

**Affiliations:** 1 Animal Health Science, R&D Centre, Natural Remedies Private Limited., Bangalore, Karnataka - 560 100, India; 2 R&D Biology, R&D Centre, Natural Remedies Private Limited., Bangalore, Karnataka - 560 100, India; 3 Formulation & Development, R&D Centre, Natural Remedies Private Limited., Bangalore, Karnataka - 560 100, India

**Keywords:** Choline Deficiency Model, Chickens, Polyherbal Formulation, Choline Chloride

## Abstract

**Objective:**

The study was designed to establish choline deficiency model (CDM) in broilers for evaluating efficacy of polyherbal formulation (PHF) in comparison with synthetic choline chloride (SCC).

**Methods:**

A total of 2,550 one-day-old Cobb 430 broiler chicks were randomly assigned to different groups in three experiments. In experiment 1, G1 and G2 served as normal controls and were fed a basal diet with 100% soybean meal (SBM) as a major protein source supplemented with and without SCC, respectively. In G3, G4, G5, and G6 groups, SBM was replaced at 25%, 50%, 75%, and 100% by soy protein isolate (SPI) to induce a graded level of choline deficiency. In experiment 2, PHF (500 and 1,000 g/ton) in comparison with SCC (1,000 g/ton) were evaluated. In experiment 3, dose-response of PHF (200, 400, and 500 g/ton) with SCC (400 g/ton) was determined.

**Results:**

Replacement of SBM by SPI produced a linear decrease in body weight gain (BWG) with a poor feed conversion ratio (FCR). 25% SBM replacement by SPI yielded an optimum negative impact on BWG and FCR; hence, it is considered for further studies. In experiment 2, PHF (500 and 1,000 g/ton) and SCC (1,000 g/ton) showed a similar performance in BWG, FCR and relative liver weight. In experiment 3, PHF produced an optimum efficacy at 400 g/ton and was comparable to SCC in the restoration of serum aspartate aminotransferase activity, abdominal fat, breast muscle lipid content and liver histopathological abnormalities.

**Conclusion:**

Replacement of SBM by SPI caused choline deficiency characterised by worsening of BWG, FCR, elevation in liver enzymes and histopathological changes indicating fatty liver. CDM was found valid for evaluating SCC and PHF. It is concluded that PHF has the potential to mimic biological activities of SCC through the restoration of negative effects caused by CDM.

## INTRODUCTION

Choline, a rediscovered vitamin B_4_ that mostly exists in the form of phospholipids, plays a critical role in several biological functions in poultry. It is essential for building and maintaining cell membranes and organelles, such as mitochondria and microsomes, and for normal maturation of the cartilage matrix of the bone [1]. Moreover, it is an essential component of acetylcholine, the most common neurotransmitter in the nervous system involved in the transmission of nerve impulses across synapses [2]. The prominent structural feature of choline is its biologically active methyl groups through which it plays a vital role as a labile methyl donor in the formation of methionine from homo-cysteine after being oxidised to betaine [3]. Furthermore, choline is considered as a lipotropic factor preventing abnormal accumulation of lipid and development of fatty liver [4].

Unlike other vitamins, choline can be synthesised through *de novo* synthesis, but the inability to synthesise a sufficient amount can cause choline deficiency, resulting in growth retardation and perosis in young chicks. Moreover, the bioavailability of native choline varies largely and depends on raw material sources and bird-related factors such as type, strain, age, feed consumption, dietary crude protein, and methionine [5–7]. In addition, the supplementation of dietary methionine or other methyl donors cannot completely replace the choline requirement of chicks as avian species have limited capacity to carry out the initial step in choline biosynthesis [8], *i.e.*, methylation of aminoethanol to methyl aminoethanol, which is contradictory to the situation with growing mammals such as pigs or rats [9]. Hence, choline has become an indispensable feed additive in the ration of broiler chickens as the above-stated problems can be counteracted by adding appropriate levels of synthetic choline chloride (SCC).

Choline chloride, a typical form of choline added to the animal diets, has some disadvantages such as high hygroscopicity, acceleration of oxidative loss of vitamins, and the formation of trimethylamine (TMA) in the gastrointestinal tract of the birds [10]. TMA is a short-chain aliphatic amine formed from dietary choline in a reaction catalysed by bacterial enzymes within the gut [11]. It is found in high concentrations in fish and is responsible for the characteristic odour of seafood [12]. These drawbacks can affect the production of organic poultry [13], and hence, the use of SCC in organic farming practices has been investigated [14]. Due to the importance of choline in poultry nutrition and production, the researchers have reinvestigated and explored the alternatives for synthetic choline from natural sources. To counteract the drawbacks of SCC addition, researchers have also focused on the addition of herbal preparations in broiler rations. A various natural products and medicinal plants, including crude extracts and compounds isolated from plants, have been utilised as an alternative to SCC in animal diets. Many researchers have reported that these herbs can mimic the choline-like activity in poultry [15–18].

The polyherbal formulation (PHF), indexed as Kolin Plus (M/s Natural Remedies Pvt Ltd, Bengaluru, India), is a combination of *Acacia nilotica* (*A. nilotica*) and *Curcuma longa* (*C. longa*) belonging to the families of Mimosaceae and Zingiberaceae, respectively. The hepatoprotective, antioxidant, and lipotropic properties of extracts of these plants have been reported individually [19–22]. However, there are no scientific data demonstrating their choline-like activities in combined form in a choline deficiency model (CDM) in poultry.

There are no appropriate models for studying choline deficiency using weight gain as the major response criterion in broiler chickens. Similarly, several attempts to evaluate the significance of choline chloride in poultry production have met with failure, as commercial ration had sufficient choline content and no difference was observed in the performance of broilers supplemented with or without SCC [23–24]. So, a suitable basal diet will allow the researchers to evaluate the problems of choline deficiency in broiler chickens. Soybean meal (SBM) is a principal source of choline that satisfies the choline requirements in poultry diets. Therefore, when intact SBM is substituted by defatted SBM (i.e., soy protein isolate [SPI]), a process to remove part of the choline, the only difference in the basal and choline-deficient diets would be the amount of choline present. This modification overcomes the problem of adding confounding factors other than choline with the test ingredient. Hence, the present study was planned to develop a CDM which could elicit the weight gain difference in broiler chickens by feeding a basal diet with SBM or SPI. Moreover, this experimentally induced CDM was used to compare the effect of newly developed PHF and SCC on growth performance, serum biochemistry, liver histopathology, and carcass traits of broiler chickens.

## MATERIALS AND METHODS

### Polyherbal formulation

Kolin Plus is a proprietary PHF developed by M/s. Natural Remedies Pvt Ltd, Bengaluru, India, containing *A. nilotica* and *C. longa* plant parts.

### Ethical approval

The study was conducted by authorised, qualified and trained veterinarians, scientists and technicians in compliance with the guidelines laid down by the Institutional Animal Ethics Committee (IAEC) approved by the Committee for the Purpose of Control and Supervision of Experiments on Animals (CPCSEA), India. All applicable international, national, and/or institutional guidelines for the care and use of animals were followed.

### Experimental setup

All three experiments were conducted in poultry research station recognised by the Department of Scientific and Industrial Research, India; DSIR Reg No.: TU/IV-RD/2000/2016, located in Anniyalam, Tamil Nadu, for a period of 40 to 42 days.

The chicks were housed in a semi-closed house divided into pens with floor space of 2.32 square meters. The approximate size of the individual pen was 1.3 m×1.8 m×0.6 m (length× width×height). Each pen was equipped with a brooder, a bell drinker, chick feeder and/or jumbo feeder. The size and floor space of the pen were modified according to the number of chicks housed with the help of a PVC sheet. House temperature was maintained at 32°C to 34°C from day 0 to 7 and then progressively reduced by 2°C weekly till week 6. House relative humidity was maintained between 40% and 70% throughout the study period. Lighting was provided 24 h daily during the first 3 days, 23 h light and 1 h darkness between days 4 and 7 and 20 h light and 4 h of darkness till day 42 [25]. The chicks were vaccinated against Marek’s disease immediately after hatching.

Upon arrival, the chicks were provided with 4% sugar-added water for the first four hours to replenish the depleted energy and stimulate feed intake. Then the chicks were individually weighed, tagged with a wing band bearing an identification number. The chlorinated potable drinking water (Innoclean, Natural Remedies Pvt Ltd., 1 tablet/500 L) and poultry mash feed manufactured as per NRC [5] by Higain Feeds & Farms India Pvt Ltd., Bangalore were provided *ad libitum* (Tables 1, 2). All birds were vaccinated against Newcastle disease (LaSota strain) and infectious bursal disease by eye drops on days 5 and 14, respectively [25].

### Experimental design and feeding levels

#### Experiment 1. Development of CDM in broilers

Nine hundred 1-day-old male Cobb 430 broiler chicks were randomly assigned to six groups with 5 replicates having 30 birds each on the first day of the experiment. G1 and G2 served as normal controls and fed basal diet supplemented with and without SCC, respectively, and 100% SBM was offered as a major protein source. In groups G3, G4, G5, and G6, SBM was replaced as a protein source at 25%, 50%, 75%, and 100% by SPI. The diet of all six groups was formulated to meet the NRC [5] requirements for all nutrients except the choline levels. The replacement of SBM by SPI at various levels was employed to induce a graded level of choline deficiency in chickens (Table 3).

#### Experiment 2. Comparative evaluation of PHF and SCC in broiler CDM

Seven hundred and fifty 1-day-old male Cobb 430 broiler chicks were randomly assigned to five groups with 5 replicates having 30 birds each on the first day of the experiment. This experiment was conducted to evaluate the efficacy of PHF at two different dose levels (500 and 1,000 g/ton feed) in comparison with SCC at a standard dose of 1,000 g/ton feed (Table 3).

#### Experiment 3. Dose-response evaluation of PHF in broiler CDM

Nine hundred 1-day-old male Cobb 430 broiler chicks were randomly assigned to six groups with 5 replicates having 30 birds each on the first day of the experiment. The objectives of this experiment were to determine the dose-response of PHF, examine the reproducibility of results of PHF at 500 g/ton and verify whether doses lesser than 500 g/ton would produce an efficacy similar to SCC (Table 3).

### Assessment of zootechnical parameters

In all three experiments, the chicks in the individual pen were observed for mortality, three times a day throughout the experimental period. The body weight (BW) of individual birds was recorded on day 1 and thereafter on days 21 and 40 or 42. Feed consumption was calculated by subtracting the amount of leftover feed from the total amount of feed offered per replicate in a group, and it was measured on days 21 and 40 or 42 [25]. Feed conversion ratio (FCR) was calculated as feed intake divided by BW. In experiments 1 and 2, liver samples were collected and weighed from 10 chickens in each group, and then the relative liver weight (RLW) was calculated as follows: (Liver weight in kg/live weight in kg)×100. However, based on the results of experiment 3, sample collection and carcass traits assessment were carried out only in groups G1, G2, G3, and G5 (n = 6) on day 40. Blood samples were collected in vacutainer tubes through the brachial vein puncture. Tissue samples (breast muscle and abdominal fat) were collected in the self-sealing polyethylene bag and stored at −80°C. Liver samples were blotted with tissue paper to remove blood and preserved in 10% neutral buffered formalin.

### Assessment of carcass traits

Carcasses were defeathered and eviscerated, and liver, heart, gizzard, and abdominal fat were immediately weighed. Cleaned carcasses (without head, neck, and feet) and parts (breast, thighs+drumsticks, and wings) were then weighed, and all the values were expressed as mean of 6 animals.

### Determination of serum aspartate aminotransferase activity

Blood samples were centrifuged at 3,000 RPM for 5 minutes, and serum was separated. In accordance with the protocol of an aspartate aminotransaferase (AST) Test Kit (Span Diagnostic Ltd., Surat, Gujarat, India), 20 μL of samples and 200 μL of the AST reagent were added in a micro-well plate, mixed thoroughly and aspirated immediately for measurement. The absorbance was read after 60 seconds, and thereafter, reading was repeated every 30 seconds up to 120 seconds at 340 nm. The mean absorbance was determined per minute, and the AST activity (IU/L) was calculated as follows: Mean absorbance×1,768 (kinetic factor).

### Determination of cholesterol level

A piece of breast muscle and abdominal fat (weighed about 250 mg) were homogenised in 2,500 μL of 10 mM phosphate-buffered saline (PBS) and then centrifuged at 1,600 g force for 10 minutes at 40°C; the supernatant was used directly in the assay. In accordance with the protocol of a Cholesterol Test Kit (Span Diagnostic Ltd., India), 2.5 μL of samples or reagent 2 (standard) was added to separate micro-well, and 250 μL of the total cholesterol reagent was added, mixed thoroughly and incubated at 37°C for 10 minutes. The absorbance was read at 505 nm and then cholesterol concentration (mg/dL) was calculated as follows: (Test absorbance/standard absorbance) ×200.

### Determination of triglyceride level

A piece of breast muscle and abdominal fat (weighed about 250 mg) were homogenised in 2,500 μL of 10 mM PBS buffer and centrifuged at 1,600 g force for 10 minutes at 40°C; the supernatant was used directly in the assay. In accordance with the protocol of a Triglyceride Test Kit (Span Diagnostic Ltd., India), 2.5 μL of samples or reagent 2 (standard) was added to a separate micro-well, and 240 μL of the total cholesterol reagent was added, mixed thoroughly and incubated at 37°C for 10 minutes. The absorbance was read at 505 nm, and the triglyceride concentration (mg/dL) was calculated as follows: (Test absorbance/standard absorbance)×200.

### Liver histopathology

Formalin-fixed liver samples were dehydrated in the series of ascending grades of alcohol (70%, 80%, 95%, and 100%), cleared in chloroform, impregnated with paraffin, and embedded in paraffin wax with ceresin. Finally, the paraffin blocks were sectioned at 4 μm thickness using a sliding microtome (Leica, Wetzlar, Germany). After sectioning, the sections were de-paraffinized in xylol followed by hydration in descending grades of alcohol (100%, 95%, 80%, and 70%) and distilled water. The sections were then stained with standard Haematoxylin and Eosin method and then mounted (DPX mountant, S.d. fine-chem Ltd., Bengaluru, Karnataka, India). The slides were then observed under a microscope (Olympus Corporation, Tokyo, Japan) connected with the camera (DP20).

### Statistical analysis

Upon completion of the trial, the raw data were compiled, processed and expressed as mean. The generated data were analysed using one-way analysis of variance technique with replicate as a blocking factor to see whether blocking was effective at reducing the random error. In case of significant differences among treatments (p<0.05), the means were subjected to least significance difference test (IBM SPSS Statistics Version.21.0; SPSS Inc., Chicago, IL, USA) [26] to draw a comparison between the groups for each investigational parameter. The value p<0.05 was considered as statistically significant.

## RESULTS

### Experiment 1: Development of CDM in broilers

The effect of feeding a basal diet containing SPI in-place of SBM as a protein source on zootechnical parameters of broiler chickens was evaluated and is presented in Table 4. Initial live BW among the groups was similar, indicating the homogeneity in the weight of birds used in the study. There was no noticeable difference observed in the performance of broilers between normal control with SCC and normal control without SCC. However, BW, feed intake and FCR were linearly decreased as the replacement of SBM by SPI as a protein source was gradually increased over the course of the experiment. CDM caused a gradual and significant (p<0.05) drastic reduction in BW and body weight gain (BWG) at 21 and 42 days of age. Similarly, feed intake was significantly decreased in all groups at 21 days of age, whereas the significant reduction was observed only in G4, G5, and G6 groups at 42 days of age when compared to G1 and G2. Besides, FCR also worsened in a dose-dependent manner with G6 and G5 showing the highest FCR (significant, p<0.05) followed by G4 and G3 at 21 and 42 days of age. Similarly, the RLW was found to have gradually increased in all groups, but a significant (p<0.05) increase was observed in the group fed with the basal diet containing 50% SBM+50% SPI and 25% SBM+75% SPI as a source of protein. In contrast, survivability was not affected in birds fed basal diet formulated with different levels of replacement of SBM by SPI.

### Experiment 2: Comparative evaluation of PHF and SCC in broiler CDM

The effect of PHF and SCC on zootechnical parameters of broiler chickens is presented in Table 5. BW and BWG were significantly increased in chicks reared on CDM and concomitantly supplemented with 1,000 g/ton of PHF, whereas only a numerical improvement in BWG was observed in 500 g/ton of PHF and 1,000 g/ton of SCC treated groups. FCR was significantly (p<0.05) decreased in all treatment groups at 21 days of age. However, all the dietary levels of PHF (500 and 1,000 g/ton) and SCC (1,000 g/ton) significantly (p<0.05) improved BW, BWG, and FCR at 42 days of age. As expected, no significant (p>0.05) differences were observed in mortality rate between the groups, and this finding was in agreement with that of experiment 1.

### Experiment 3: Dose-response evaluation of PHF in broiler CDM

Dose-response of PHF on zootechnical parameters is presented in Table 6. PHF (400 g/ton) was found to produce a significantly higher BW and BWG when compared to other treatment groups, whereas BW and BWG in PHF (200 and 500 g/ton) were similar to SCC (400 g/ton) group. FCR was significantly (p<0.05) improved in PHF (200, 400, and 500 g/ton) group while FCR was numerically decreased in SCC (400 g/ton) group at 40 days of age. In contrast, no significant (p>0.05) differences were observed in survivability and feed intake among various treatment groups.

**Figure 1:**
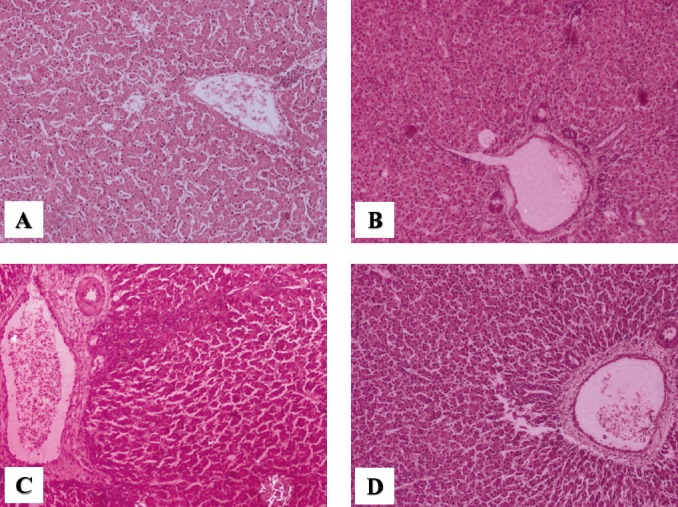
Photomicrographs (optical microscopy) of haematoxylin and eosin-stained broiler liver sections from different treatments. Normal liver architecture in control (A) animals and mild vacuolar changes in hepatocytes of poly herbal formulation (D) treated chickens were observed. Obvious liver lesion such as moderate cell swelling, decreased sinusoidal spaces and mild vacuolar degeneration in hepatocytes were observed in choline deficiency model (B). Mild cell swelling and vacuolar changes with reduced sinusoidal spaces in hepatocytes were visible in synthetic choline chloride (C) treated chickens. Original magnification: 10× (n = 4).

CDM caused a significant elevation in the levels of triglyceride and cholesterol in both abdominal fats (p<0.05) and breast muscle. However, PHF (400 g/ton) was found to decrease the levels of triglyceride and cholesterol in both abdominal fat (p< 0.05) and breast muscle while the levels of triglyceride and cholesterol (p<0.05) were decreased only in the breast muscle of SCC (400 g/ton) supplemented birds (Table 7). Correspondingly, SCC and PHF (400 g/ton) treated groups displayed a significant (p<0.05) improvement in serum AST activity that was enhanced by CDM, yet the best reduction was accomplished in G3 followed by G5. In contrast, SCC or PHF supplement in CDM had no significant (p>0.05) effect on carcass yield (Table 7). The histopathological studies revealed a moderate cell swelling with decreased sinusoidal spaces in hepatocytes of birds reared on CDM. In contrast, reduced cell swelling and mild vacuolar changes were observed in hepatocytes of birds supplemented with SCC or PHF (400 g/ton) (Figure 1).

## DISCUSSION

Choline is a well-recognised nutrient that prevents fatty liver, perosis and growth retardation in poultry. Dietary methionine can provide methyl groups for *de novo* synthesis of choline, but young chicks have limited ability to perform the first methylation of phosphatidylethanolamine because S-adenosyl-methionine is inefficient as a methyl donor in choline biosynthesis [8,27]. Irreversible oxidation of choline to betaine also prevents the betaine from serving as a choline precursor. Methionine and betaine supplementation can replace one function of choline, namely, provision of a methyl group to the single-carbon pool. However, choline is also required for acetylcholine and phospholipid synthesis, which are important than methyl donation as indicated by previous research works that showed no response to betaine in a choline-free purified diet until two-thirds of the choline requirement have been furnished by choline *per se* [28]. Thus, it can be said that a diet severely deficient in choline will not elicit a growth response upon addition of methionine or betaine, whereas a diet marginally deficient in choline will probably respond to either of these compounds [29]. This shows the significance of the dietary inclusion of choline in poultry diets.

As per the NRC [5] recommendation, choline requirements of broilers for weight gain and feed intake were 1,472 and 1,424 mg/kg during starter and finisher stages, respectively. Previous studies have determined and confirmed choline requirement and availability of choline in feedstuffs using corn starch and isolated soybean protein diet [30–32]. However, no specific experiments have been conducted to evaluate the problems of choline deficiency in broiler chickens, *viz*., growth retardation, worsening of FCR and fatty liver. Moreover, several attempts to evaluate the significance of choline chloride in poultry production have met with failure as corn-SBM diet fulfils the choline requirements of broilers supplemented with or without SCC [5]. As it is apparent to have an appropriate experimental diet to elicit the growth response upon supplementation of choline, SBM was replaced by SPI as a protein source at various levels in poultry ration, and its impact on zootechnical parameters was evaluated in experiment 1.

Similar performance in normal controls with and without SCC indicates that the endogenous choline requirement was fulfilled by major sources of feedstuffs, *i.e.*, corn and SBM during normal conditions. This finding was supported by Briz and Perez [33] who reported that the corn and soybean-based diets contain approximately 1,350 mg choline/kg and do not require choline supplementation in diet [34], even at low dietary methionine levels [35]. However, CDM depressed the total BWG by 6%, 17%, 40.1%, and 71.4% and increased the feed consumption to 110 g, 160 g, 240 g, and 1,200 g, respectively, feed per unit BWG of broiler chicken raised on diets prepared using SPI (25%, 50%, 75%, and 100%) in place of SBM (75%, 50%, 25%, and 0%) as a protein source. Severe weight loss, as well as worst FCR, was observed in the groups with 100%, followed by 75%, 50%, and 25% replacement of SBM with SPI (as a protein source). This linear decline in weight gain, feed efficiency and increase in RLW to increasing levels of SBM replacement with SPI as a protein source in diets suggests that choline deficiency was experimentally induced in a dose-dependent manner, which in-turn negatively influenced the production parameters of broiler chickens. This observation was in accordance with the reports by Giovani et al [29], Ryan et al [30] and, Emmert and Baker [31], who found an almost linear response to the incremental supplementation of choline chloride in chicks fed choline-deficient basal diet, indicating the positive role of supplemental choline on weight gain of birds. Besides, perosis and growth depression have been reported as the characteristic symptoms of choline deficiency in ducks [35]. The results of the present study further confirmed that growth retardation and worsening of FCR could be considered as reliable and sensitive measures of choline deficiency in broiler chickens.

Based on the results from experiment 1, 25% replacement of SBM by SPI was selected for further screening of PHF in comparison with SCC as it yielded an optimum negative impact on BWG and FCR through optimum induction of choline deficiency in broilers. Hence, 100% SBM without SCC and 25% replacement of SBM by SPI as normal and negative controls, respectively, were used in experiments 2 and 3. The observed lower BWG (6.3% and 3.9%) with higher feed consumption per unit BWG (90 and 44 g) in the negative control group of experiments 2 and 3 were correlated with the results of experiment 1 as expected.

In experiment 2, two different dose levels of PHF were evaluated and compared with 60% of SCC (1,000 g/ton) as the choline requirement of young chicks consuming a purified diet was previously shown to be 600 mg/kg [30,31]. CDM supplemented with SCC (1,000 g/ton) or PHF (500 and 1,000 g/ton) independently improved the weight gain by about 5.4%, 6.5%, and 4.3%, respectively, and reduced the feed consumption, *viz.*, 65 g, 82 g, and 32 g, respectively, feed per unit BWG. However, the response to PHF (500 g/ton) was greater than SCC and PHF (1,000 g/ton) and was comparable to the normal control response. Based on the previous experimental results, experiment 3 was performed to determine the dose-response of PHF (200, 400, and 500 g/ton) and verify whether doses lesser than 500 g/ton would produce the efficacy similar to SCC as well as the reproducibility of PHF at 500 g/ton dose. Moreover, 400 g/ton of SCC (60%) was used as a positive control because the difference in choline concentration between normal and negative diets was calculated as 400 mg/kg. SCC (400 g/ton) or PHF (200, 400, and 500 g/ton) displayed 2.1%, 1.3%, 4.1%, and 1.7% weight gain improvement respectively, and consumed 32 g, 40 g, 68 g, and 60 g, respectively, less feed per unit BWG. However, greater performance was observed in PHF (400 g/ton) group when compared to other groups. This response signifies that PHF could effectively replace the SCC from broiler ration at the dose of 400 g/ton, which performs a similar function of SCC as reflected from the similar performance index. This was in accordance with the findings of Khosravinia et al [38], Arele et al [1], Chatterjee and Misra [15], Muthukumarasamy et al [16], Kumar [39], and Yu [40] who confirmed that weight gain, feed intake, feed conversion and viability of broiler chickens were similar or improved when the choline chloride was replaced by a vegetal source of choline in the diets. In addition, low RLW in PHF or SCC groups indicates liver protection and normal function; this was supported by Harms and Russell [40], who reported that choline chloride supplementation in diets decreases the liver, spleen and heart weights in broiler chickens.

A change in carcass percentage was anticipated as increased abdominal and breast muscle fats were observed in experiment 3. However, no difference was observed in carcass yield of the birds fed on diets with SCC or PHF. These results are in accordance with the findings of Khosravinia et al [38], who reported an improvement in BW and FCR but no change in carcass yield percentage when broilers were fed moderate and high energy diets supplemented with Bio choline, choline chloride and lecithin extract.

Choline is a well-known lipotropic factor responsible for the mobilisation of liver fat in the form of lipoproteins toward extra-hepatic tissues where they may be metabolised or deposited [42,43]. As a result, the excess fat energy gets diverted towards muscle protein accretion rather than body fat synthesis, resulting in better growth, improved FCR and lower lipid content in liver, abdomen, and carcass [37]. The reduction in lipid content observed in the present study could be attributed to the lipotropic effect of PHF (abdominal fat and breast muscle) and SCC (breast muscle), yet PHF (400 g/ton) was more potent as having the hypo-cholesterolemic effect than SCC (400 g/ton). This corroborates with the findings of Devegowda et al [44] who observed that fat was decreased in the abdomen and liver in broilers supplemented with an herbal source of choline. However, SCC did not reverse lipid content to normal in abdominal fat. This might be due to the insufficient dose of SCC (400 g/ton), and further investigation is required to evaluate the effect of SCC level on this parameter.

Liver, a principal organ in avian metabolism, is susceptible to nutritional alterations. The liver health and functionality are assessed based on serum alanine transaminase (ALT) and AST enzyme activity, as the cell damages caused by metabolic pressure and hypertension ease the liberation of these cellular enzymes into the serum [45]. In experiment 3, the hepatic damage was confirmed through high serum AST enzyme activity and histopathological changes in birds fed with choline-deficient diet while it was reversed in the birds supplemented with PHF or SCC; this demonstrates the role of choline in liver protection. This was supported by Khosravinia et al [38], who reported that choline chloride supplementation prevents hepatic damage as evidenced by low AST level in serum.

The PHF used in the current study contains *A. nilotica* and *C. longa*, which are a rich source of polyphenols and curcuminoids, respectively, and can mimic the hepatoprotective activity of choline. This statement was supported by Narayanan Kannan et al [19], who proved the protective effect of *A. nilotica* on acetaminophen-induced hepatotoxicity wherein pre-treatment with *A. nilotica* (250mg/kg) orally in rats attenuated the liver damage and enhanced serum activities of ALT, AST, alkaline phosphatase, liver weight and total bilirubin levels caused by administration of acetaminophen. Similarly, Alli [46] demonstrated that there was a significant decrease in total cholesterol and triglycerides at 500 mg/kg of *A. nilotica* in both male and female rats. In addition, Tranchida et al [47] reported that the supplementation of *C. longa* extracts caused an impact on transmethylation pathway and/or osmotic regulation by increasing the liver betaine content, which plays a role in liver lipid metabolism. These findings strongly indicate that improvement in zootechnical parameters (BW and FCR) and alleviation of AST activity, liver histopathology and lipid content (abdominal fat and breast muscle) of the PHF supplemented group to normal could be attributed to its hepatoprotective and lipotropic activity. These research findings confirm that PHF possesses choline-like activities and performs at par with SCC; hence, it could be used as a natural replacer and greener alternative to SCC in animal diets.

## CONCLUSION

In conclusion, choline deficiency could cause growth depression in meat broiler chickens. In this study, CDM was successfully induced and established using 75% SBM+25% SPI as a source of protein, which is suitable to measure the characteristic symptom of choline deficiency, *i.e.*, growth performance in broiler chickens. CDM was found valid in the screening of products possessing choline-like activities. Also, it was confirmed that PHF has the potential to replace the function of 1 kg/ton of synthetic choline (choline chloride 60%) at 400 g/ton inclusion rate in broiler diets; this was reflected by the improved growth performance and feed efficiency.

## Figures and Tables

**Table 1:** Experiment 1: Ingredients and chemical composition of starter feed (g)

Ingredients	G1	G21)	G31)	G4	G5	G6
Maize	620.00	621.00	620.00	635.00	640.00	647.00
Deoiled rice bran	0.00	0.00	42.15	68.65	96.90	129.30
Soybean meal	294.00	294.00	220.50	147.00	73.50	0.00
Soy protein isolate	0.00	0.00	42.30	84.50	126.80	169.10
Calcite	13.00	13.00	13.00	13.00	14.00	14.00
DCP	5.00	5.00	5.00	5.00	5.00	5.00
Oil	15.00	15.00	15.00	15.00	17.00	18.00
Maize gluten meal	40.00	40.00	30.00	20.00	15.00	6.00
Methionine	2.40	2.40	2.40	2.40	2.40	2.40
Lysine	2.50	2.50	2.30	2.10	1.80	1.60
Threonine	0.90	0.90	0.90	0.90	0.90	0.90
Betaine	0.50	0.50	0.50	0.50	0.50	0.50
NSP enzyme	0.50	0.50	0.50	0.50	0.50	0.50
Phytase 2500	0.20	0.20	0.20	0.20	0.20	0.20
Sodium bicarbonate	1.00	1.00	1.00	1.00	1.00	1.00
Salt	2.00	2.00	2.00	2.00	2.00	2.00
Potassium chloride	0.00	0.00	0.25	0.25	0.50	0.50
Vitamin premix2)	0.50	0.50	0.50	0.50	0.50	0.50
Choline chloride 60%	1.00	0.00	0.00	0.00	0.00	0.00
AGP	0.50	0.50	0.50	0.50	0.50	0.50
Coccidiostat	0.50	0.50	0.50	0.50	0.50	0.50
TM premix3)	1.00	1.00	1.00	1.00	1.00	1.00
Nutrient composition (calculated value)						
CP (%)	21.10	21.11	21.09	20.98	21.12	21.09
ME (Mcal/kg)	3.12	3.12	3.10	3.10	3.10	3.10
Lysine (%)	1.22	1.22	1.21	1.20	1.19	1.18
Methionine (%)	0.69	0.69	0.69	0.68	0.69	0.69
Threonine (%)	0.87	0.87	0.87	0.87	0.88	0.88
Calcium (%)	0.78	0.78	0.78	0.77	0.80	0.79
Available phosphorus (%)	0.41	0.41	0.40	0.40	0.40	0.40
Choline (ppm)	1,655.29	1,205.90	1,036.83	863.52	687.29	514.70

DCP, dicalcium phosphate; NSP, non-starch polysaccharide; AGP, antibiotic growth promoters; TM, trace minerals; CP, crude protein; ME, metabolisable energy.

1) G2 & G3 feed were used for normal and choline deficiency model control group in experiment 2 and 3.

2) The vitamin premix supplied the following per kilogram of vitamin premix: vitamin A, 25 MIU; vitamin D_3_, 5 MIU; vitamin E, 24 IU; vitamin K, 3 g; vitamin B_1_, 3 g; vitamin B_2_, 10 g; vitamin B_6_, 4 g; vitamin B_12_, 0.015 g; niacin, 30 g; pantothenic acid, 20 g; folic acid, 1g.

3) The mineral premix supplied the following per kilogram of TM premix: Fe, 40 g; Cu, 10 g; Mn, 100 g; Zn, 100 g; Se, 0.25 g; and I, 1.5 g.

**Table 2:** Experiment 1: Ingredients and chemical composition of finisher feed (g)

Ingredients	G1	G21)	G31)	G4	G5	G6
Maize	665.20	666.00	671.00	674.00	684.00	689.30
Deoiled rice bran	0.00	0.00	26.10	53.90	74.20	100.00
Soybean meal	235.30	235.00	176.30	117.50	58.80	0.00
Soy protein isolate	0.00	0.00	33.80	67.60	101.30	135.10
Calcite	12.00	12.00	12.00	12.00	12.00	12.00
DCP	3.00	3.00	3.00	3.00	4.40	4.00
Meat and bone meal	10.00	10.00	10.00	10.00	8.00	8.00
Oil	25.00	25.00	26.00	27.00	27.00	28.00
Maize gluten meal	36.00	36.00	29.00	22.50	18.00	11.50
Methionine	2.00	2.00	2.00	2.00	2.00	2.00
Lysine	2.70	2.70	2.60	2.30	2.20	2.00
Threonine	1.20	1.20	1.10	1.10	1.00	1.00
Betaine	0.50	0.50	0.50	0.50	0.50	0.50
NSP enzyme	0.50	0.50	0.50	0.50	0.50	0.50
Phytase 2500	0.20	0.20	0.20	0.20	0.20	0.20
Sodium bicarbonate	0.75	0.75	0.75	0.75	0.75	0.75
Salt	2.20	2.20	2.20	2.20	2.20	2.20
Potassium chloride	0.00	0.40	0.40	0.40	0.40	0.40
Vitamin premix2)	0.50	0.50	0.50	0.50	0.50	0.50
Choline chloride 60%	1.00	0.00	0.00	0.00	0.00	0.00
AGP	0.50	0.50	0.50	0.50	0.50	0.50
Coccidiostat	0.50	0.50	0.50	0.50	0.50	0.50
TM premix3)	1.00	1.00	1.00	1.00	1.00	1.00
Nutrient composition (calculated value)						
CP (%)	19.00	18.99	18.98	19.00	19.01	19.01
ME (Mcal/kg)	3.22	3.23	3.22	3.22	3.22	3.22
Lysine (%)	1.10	1.10	1.09	1.08	1.07	1.06
Methionine (%)	0.62	0.62	0.62	0.62	0.62	0.62
Threonine (%)	0.89	0.89	0.89	0.89	0.89	0.89
Calcium (%)	0.81	0.81	0.80	0.79	0.76	0.78
Available phosphorus (%)	0.399	0.399	0.397	0.396	0.393	0.401
Choline (ppm)	1,538.37	1,088.14	950.11	812.36	670.20	531.00

DCP, dicalcium phosphate; NSP, non-starch polysaccharide; AGP, antibiotic growth promoters; TM, trace minerals; CP, crude protein; ME, metabolisable energy.

1) G2 and G3 feed were used for normal and choline deficiency model control group in experiment 2 and 3.

2) The vitamin premix supplied the following per kilogram of vitamin premix: vitamin A, 25 MIU; vitamin D_3_, 5 MIU; vitamin E, 24 IU; vitamin K, 3 g; vitamin B_1_, 3 g; vitamin B_2_, 10 g; vitamin B_6_, 4 g; vitamin B_12_, 0.015 g; niacin, 30 g; pantothenic acid, 20 g; folic acid, 1 g.

3) The mineral premix supplied the following per kilogram of TM premix: Fe, 40 g; Cu, 10 g; Mn, 100 g; Zn, 100 g; Se, 0.25 g; and I, 1.5 g.

**Table 3:** Study design

Groups	Major protein source		SCC (g/ton feed)	

	SBM (%)	SPI (%)		
Experiment 1: Effect of CDM on zootechnical parameters in Cobb 430 broiler chickens				
G1	100	0	1,000	
G2	100	0	0	
G3	75	25	0	
G4	50	50	0	
G5	25	75	0	
G6	0	100	0	

**Groups**	**Major protein source**		**SCC (g/ton feed)**	**PHF (g/ton feed)**

	**SBM (%)**	**SPI (%)**		

Experiment 2: Comparative evaluation of PHF and SCC in Cobb 430 broiler CDM				
G1, Normal control	100	0	0	0
G2, CDM control	75	25	0	0
G3, CDM+SCC	75	25	1,000	0
G4, CDM+PHF	75	25	0	500
G5, CDM+PHF	75	25	0	1,000
Experiment 3: Dose-response evaluation of PHF in Cobb 430 broiler CDM				
G1, Normal control	100	0	0	0
G2, CDM control	75	25	0	0
G3, CDM+SCC	75	25	400	0
G4, CDM+PHF	75	25	0	200
G5, CDM+PHF	75	25	0	400
G6, CDM+PHF	75	25	0	500

CDM, choline deficiency model; SBM, soybean meal; SPI, soy protein isolate; SCC, synthetic choline chloride; PHF, polyherbal formulation.

**Table 4:** Experiment 1: Effect of CDM on zootechnical parameters in Cobb 430 broiler chickens

Parameters	Day	NC+SCC	NC–SCC	75% SBM+ 25% SPI–SCC	50% SBM+ 50% SPI–SCC	25% SBM+ 75% SPI–SCC	0% SBM+ 100% SPI–SCC	Pooled SEM	p value
BW (g), (n = 5)	1	42	43	42	42	42	41	0.2	0.072
	21	717^a^	719^a^	667^b^	536^c^	279^d^	148^e^	41.4	0.000
	42	2,334^a^	2,360^a^	2,219^b^	1,958^bc^	1,413^bd^	674^be^	116.2	0.000
BWG (g), (n = 5)	21	675^a^	676^a^	625^b^	494^c^	237^d^	107^e^	41.3	0.000
	42	2,292^a^	2,317^a^	2,177^b^	1,916^bc^	1,371^bd^	633^be^	116.1	0.000
FCR (n = 5)	21	1.31^a^	1.31^a^	1.33^a^	1.44^a^	1.68^b^	1.83^c^	0.03	0.000
	42	1.57^a^	1.58^a^	1.69^a^	1.74^a^	1.82^a^	2.78^b^	0.02	0.000
Feed intake (g), (n = 5)	21	937^a^	943^a^	885^b^	771^c^	469^d^	270^e^	36.5	0.000
	42	3,659^a^	3,732^a^	3,745^a^	3,392^b^	2,555^c^	1,610^d^	94.5	0.000
Mortality (%)		2.0	1.3	2.7	1.3	3.3	2.7	-	-
Relative liver weight (g) (n = 10)		1.6^a^	1.6^a^	1.8^ab^	1.9^b^	2.0^b^	2.3^c^	0.03	0.000

CDM, choline deficiency model; NC, normal control; SCC, synthetic choline chloride; SBM, soybean meal; SPI, soy protein isolate; SEM, standard error of the mean, BW, body weight; FCR, feed conversion ratio.

a–e Means bearing different superscripts in a row differ significantly (p<0.05).

**Table 5:** Experiment 2: Comparative evaluation of PHF and SCC in Cobb 430 broiler CDM

Parameters	Day	NC	CDM control	CDM+SCC (1,000 g/ton)	CDM+PHF (500 g/ton)	CDM+PHF (1,000 g/ton)	Pooled SEM	p value
BW (g) (n = 5)	1	46	46	46	46	46	0.2	0.996
	21	666^ab^	645^a^	674^ab^	659^ab^	693^b^	7.1	0.295
	39	1,940^b^	1,822^a^	1,919^b^	1,937^b^	1,916^b^	12.3	0.004
	42	2,169^b^	2,035^a^	2,146^b^	2,168^b^	2,123^b^	14.5	0.008
BWG (g) (n = 5)	21	620^ab^	599^a^	627^ab^	612^ab^	647^b^	7.1	0.300
	39	1,893^b^	1,776^a^	1,873^b^	1,890^b^	1,870^b^	12.3	0.004
	42	2,122^b^	1,989^a^	2,099^b^	2,122^b^	2,077^b^	14.5	0.008
FCR (n = 5)	21	1.56^a^	1.58^a^	1.45^b^	1.47^b^	1.48^b^	0.01	0.000
	39	1.63^a^	1.72^b^	1.64^a^	1.62^a^	1.68^c^	0.01	0.000
	42	1.65^a^	1.74^b^	1.68^a^	1.66^a^	1.71^c^	0.01	0.000
Feed intake (g) (n = 5)	21	1,036^a^	1,020^a^	977^b^	971^b^	1,021^a^	8.1	0.017
	39	3,157	3,131	3,155	3,140	3,209	15.6	0.600
	42	3,582	3,545	3,597	3,599	3,631	16.6	0.620
Mortality (%)		0.7	0.7	0.00	1.3	0.00	-	-
Relative liver weight (g) (n = 10)		1.9	2.1	2.0	1.9	2.0	0.03	0.501

PHF, polyherbal formulation; SCC, synthetic choline chloride; CDM, choline deficiency model; NC, normal control; CDM, choline deficiency model; SEM, standard error of the mean; BW, body weight; FCR, feed conversion ratio.

a–c Means bearing different superscripts in a row differ significantly (p<0.05).

**Table 6:** Experiment 3: Dose-response evaluation of PHF in Cobb 430 broiler CDM

Parameters	Day	NC	CDM control	CDM+SCC (400 g/ton)	CDM+PHF (200 g/ton)	CDM+PHF (400 g/ton)	CDM+PHF (500 g/ton)	Pooled SEM	p value
BW (g) (n = 5)	1	42	41	42	41	42	42	0.2	0.691
	21	781^b^	693^a^	706^a^	684^a^	688^a^	690^a^	7.1	0.000
	40	2,134^b^	2,051^a^	2,092^ab^	2,077^ab^	2,135^b^	2,085^ab^	10.7	0.151
BWG (g) (n = 5)	21	739^b^	651^a^	663^a^	642^a^	646^a^	648^a^	7.0	0.000
	40	2,092^b^	2,010^a^	2,050^ab^	2,036^ab^	2,093^b^	2,043	10.6	0.150
FCR (n = 5)	21	1.30^a^	1.47^b^	1.45^b^	1.46^b^	1.46^b^	1.44^b^	0.02	0.000
	40	1.67^ab^	1.71^c^	1.68^ac^	1.67^ab^	1.64^b^	1.65^ab^	0.01	0.004
Feed Intake (g) (n=5)	21	1,017	1,018	1,025	1,001	1,007	995	5.6	0.533
	40	3,553	3,503	3,508	3,466	3,503	3437	18.1	0.461
Mortality (%)		0.0	0.7	0.0	0.0	1.3	2.7	-	-
Relative liver weight (g) (n = 6)		2.1	2.2	2.1	-	2.1	-	0.05	0.870

SEM, standard error of the mean; NC, normal control; CDM, choline deficiency model; SCC, synthetic choline chloride; PHF, polyherbal formulation.

a–c Means bearing different superscripts in a row differ significantly (p<0.05).

**Table 7:** Effect of PHF on carcass traits, lipid profile and liver function test in Cobb 430 broiler chickens

Parameters		NC	CDM control	CDM+SCC (400 g/ton)	CDM+PHF (500 g/ton)	Pooled SEM	p value
Carcass traits	Eviscerated carcass weight (g)	1,476	1,443	1,434	1,470	22.6	0.901
	Liver weight (g)	47	49	47	48	1.2	0.906
	Gizzard weight (g)	32	36	33	30	1.1	0.289
	Abdominal fat weight (g)	26	30	28	27	1.0	0.419
	Heart weight (g)	11	11	11	12	0.3	0.648
	Dressing (%)	70	70	69	70	0.3	0.232
	Leg weight (g)	448	472	476	462	7.6	0.562
	Wing weight (g)	150	153	154	152	2.3	0.944
	Breast weight (g)	434^a^	370^b^	372^b^	404^ab^	10.1	0.062
	Cooking loss (%)	12	13	14	13	0.5	0.538
Breast muscle fat	Triglycerides (md/dL)	2	3	2	2	0.2	0.363
	Cholesterol (md/dL)	0.7^a^	0.7^a^	0.4^b^	0.5^ab^	0.04	0.032
Abdominal fat	Triglycerides (md/dL)	11^a^	17^b^	20^c^	12^a^	0.9	0.000
	Cholesterol (md/dL)	4^a^	6^b^	8^c^	3^a^	0.4	0.000
Liver enzyme	Serum AST (IU/L)	4^a^	8^b^	4^a^	5^a^	0.6	0.012

PHF, poly herbal formulation; NC, normal control; CDM, choline deficiency model; SCC, synthetic choline chloride; SEM, standard error of the mean; n = 6; AST, aspartate aminotransaferase.

a–c Means bearing different superscripts in a row differ significantly (p<0.05).
